# CXCL8 greatly enhances neutrophil extracellular traps formation induced by calcium crystals *in vitro* and *in vivo*

**DOI:** 10.3389/fphar.2026.1794524

**Published:** 2026-04-20

**Authors:** Irene Fasciani, Maria De Lucia, Gemma Conte, Lucia Apparente, Ida Cerqua, Chayakorn Petchakup, Francesco Petragnano, Gabriella Aloisi, Giada Cremonesi, Nicola Detta, Han Wei Hou, Raffaele Capasso, Rafael Cypriano Dutra, Riccardo Bertini, Andrea Aramini, Marcello Allegretti, Roberto Maggio, Pier Giorgio Amendola, Mario Rossi

**Affiliations:** 1 Department of Biotechnological and Applied Clinical Sciences, University of L’Aquila, L’Aquila, Italy; 2 Preclinical Research, Dompé Farmaceutici S.p.a., L’Aquila, Italy; 3 Department of Pharmacy, School of Medicine and Surgery, University of Naples Federico II, Naples, Italy; 4 School of Mechanical and Aerospace Engineering, Nanyang Technological University, Singapore, Singapore; 5 Department of Agricultural Sciences, University of Naples Federico II, Naples, Italy; 6 ATREIUS S.a.s., L’Aquila, Italy; 7 R&D Pharmacon, Consulting Services in Life Sciences & Pharma, Sutri, Italy

**Keywords:** calcium aggregates, CXCL8, inflammation, NETosis model, neutrophils

## Abstract

Neutrophil extracellular traps (NETs) contribute to host defense but can also drive sterile inflammation and tissue injury. Among particulate inducers, monosodium urate and calcium crystals are potent triggers of NETosis. The role of the chemokine interleukin-8 (CXCL8) in NET formation, however, has remained controversial and context-dependent. Here we show that CXCL8 markedly amplifies NETosis induced by calcium carbonate (CaCO_3_) and calcium pyrophosphate dihydrate (CPPD) crystals in human neutrophils. This cooperative effect was independent of crystal composition, required cytoskeletal rearrangements, and was abrogated by inhibition of Src family kinases, PI3K, or ERK1/2, but not by blockade of NADPH oxidase. In a murine model, combined administration of CXCL8 and CaCO_3_ crystals significantly increased serum citrullinated histone H3 compared with crystals alone, confirming the potentiating effect *in vivo*. These findings identify an unrecognized crosstalk between chemokine signaling and crystal-driven NETosis, providing mechanistic insight into sterile inflammatory diseases including gout, pseudogout, atherosclerosis, and pancreatitis, and supporting the concept that antagonists of CXCL8 receptors (CXCR1/2) could limit pathological NET formation.

## 1 Introduction

Neutrophil extracellular traps (NET) formation is a biological process that evolved to degrade virulence factors and kill bacteria (Brinkmann et al., 2004). NETs are mesh-like structures composed of histones, DNA fibres, and various neutrophil granule proteins such as elastase, cathelicidin, cathepsin G, and myeloperoxidase (MPO). By remaining anchored to these fibres, these proteins can reach high local concentrations and exert antimicrobial activity (Huang et al., 2022). To release their DNA traps neutrophil produces toxic chemicals like NADPH oxidase-mediated reactive oxygen species (ROS) that dissolve their own internal membranes, resulting in the immediate death of the cell, this is why the process is called suicidal NETosis (Fuchs et al., 2007) In contrast, a distinct form known as vital NETosis has been described (Thiam et al., 2020; Hidalgo et al., 2022) where neutrophils remain viable. Unlike the massive release of NETs during a cell explosion in the suicidal NETosis, in vital NETosis, neutrophils eject their DNA through small bubbles or pores without using ROS. Because the outer membrane stays intact, the cell remains alive and continue to hunt and eat bacteria for several hours, even after losing their nuclear DNA, functioning like “zombie soldiers” without their brain (Huang et al., 2022; Tan et al., 2023). Beyond the canonical protective role of NET formation, recent evidence highlights its contribution to several pathological conditions, such as vessel occlusion, tissue damage, sterile inflammation, and cancer (Hidalgo et al., 2022). Therefore, there is a pressing need for effective therapies that can inhibit aberrant NET formation while preserving their antibacterial function (Singh et al., 2023).

CXCL8, a chemokine involved in inflammation and cancer, is a potent stimulator of neutrophil chemotaxis by activating the CXCR1 and CXCR2 receptors (Matsushima et al., 2022). However, its role in NETosis remains controversial. Some studies suggest that CXCL8 can directly induce NETosis (Teijeira et al., 2021), while others show that CXCL8 alone is insufficient to trigger NETosis (Branzk and Papayannopoulos, 2013), highlighting the need to clarify the contexts in which CXCL8 acts as a NETotic stimulus. Interestingly, some authors raised the possibility that CXCL8 may promote NETosis in a calcium-dependent manner, potentially through the activation of calmodulin-dependent kinases or other calcium-sensitive pathways involved in NET formation (Gupta et al., 2014; Douda et al., 2015; Hann et al., 2020). In this study, we re-evaluated the role of calcium in promoting CXCL8 induced NETosis, proposing its role within microcrystals rather than as soluble free ion. Microcrystals, formed either by calcium of other ions, have in common the ability to activate leukocytes such as neutrophil granulocytes triggering an inflammatory response (Rada, 2017; Li et al., 2018), leading to gout, pseudogout, cartilage degradation and osteoarthritis (Pascart et al., 2024) to name a few. These microcrystals are among the most potent pro-inflammatory stimuli known to activate the innate immune response, functioning as metabolite-derived damage-associated molecular patterns (DAMPs) that drive pathological inflammation (Fuchs et al., 2007; Kang et al., 2024). For instance, calcium carbonate (CaCO_3_) crystals have been shown to induce NETosis and are implicated in the pathogenesis of acute and chronic pancreatitis (Gerolami et al., 1989; Leppkes et al., 2016). An intriguing aspect of crystal-induced NET activation is that NETosis may occur independently of ROS formation, indicating the induction of a form of NETosis compatible with vital NETosis (Fuchs et al., 2007; Tan et al., 2023). Whether these microcrystals can cooperate with inflammatory chemokines such as CXCL8 to exacerbate NETosis and promote tissue damage has not been explored and will be the aim of this study.

## 2 Materials and methods

### 2.1 Culture media and reagents

Ham’s F-12K (Kaighn’s) medium, Roswell Park Memorial Institute (RPMI) 1,640 medium, Hank’s balanced salt solution (HBSS) and Sytox Green nucleic acid stain were purchased from ThermoFisher Scientific. Recombinant human interleukin-8 (CXCL8) was from Miltenyi Biotech. Calcium chloride solution; SCH527123 and inhibitors were obtained from Sigma Aldrich.

### 2.2 Calcium aggregate formation

**FIGURE 1:**
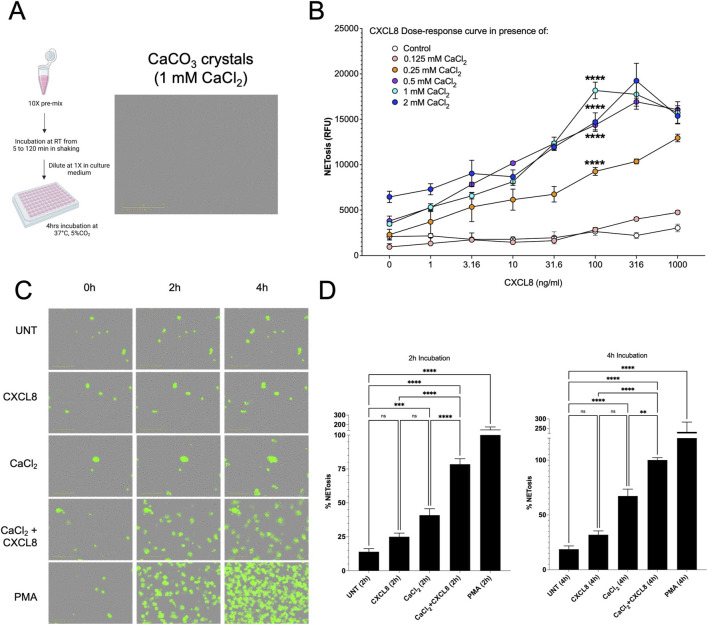
CXCL8 enhances calcium crystal-mediated NETosis. **(A)** – Left panel: Schematic representation of the protocol used to induce calcium crystal formation in 96-well plates. A pre-mix containing 10 mM CaCl_2_ was incubated for 2 h at 600 rpm and then added to the wells containing neutrophils and Sytox Green dye, reaching the final 1 mM CaCl_2_ concentration. NETosis was assessed 4 h after CXCL8 addition by measuring green fluorescence (reported as RFU) using a multiplate reader. Right panel: Representative image of calcium crystal formation within the wells, prepared with 1 mM CaCl_2_ according to the described protocol, and acquired at ×20 magnification using the Incucyte® ZOOM live-cell imaging system. **(B)** – The graph shows that CXCL8 significantly enhanced calcium crystals mediate NETs with p values lower than 0.0001 when neutrophils were treated with 0.25 mM, 0.5 mM, 1 mM, 2 mM calcium chloride two-way ANOVA followed by Tukey’s multiple comparisons test was used for statistical analysis. The statistical analysis refers to CXCL8 at 100 ng/mL, and compares its effect to the basal level of NETosis induced by each corresponding crystal suspension in the absence of CXCL8 (0 ng/mL). **(C)** – Representative images of isolated neutrophils stained with Sytox Green and treated as indicated. Images were acquired at 0, 2, and 4 h after stimulation using the Incucyte® ZOOM live-cell imaging system. Neutrophils were stimulated with 100 ng/mL CXCL8, 1 mM CaCl_2_, or their combination. PMA (10 nM) was included as positive control. **(D)** – Quantification of NET formation using the Incucyte® ZOOM Analyzer. Neutrophils were treated with 100 ng/mL CXCL8, 1 mM CaCl_2_, or their combination. NET area per well was measured at 2- and 4-h following stimulation and normalized to the condition with 100 ng/mL CXCL8 + 1 mM CaCl_2_ at 4 h (set as 100%). Ten (10) nM PMA was used as positive control. Data represents five independent experiments; each performed in triplicate. Statistical significance was determined using one-way ANOVA followed by Sidak’s multiple comparisons test.

A 1 M aqueous solution of calcium chloride (CaCl_2_) was diluted to a final concentration of 10 mM in alkalinized Ham’s F12K or RPMI1640 medium (pH 7.8–8.0) in a vial. The mixture was agitated at 600 rpm for 120 min to ensure thorough equilibration (Figure 1A). This prepared solution served as a 10× stock for experiments requiring a final CaCl_2_ concentration of 1 mM. When the 10× stock solution was diluted to a final concentration of 1 mM in the wells of a multi-well plate, formed calcium crystals were visible under ×20 magnification (Figure 1A).

### 2.3 Human neutrophil isolation

Peripheral blood from healthy adult donors was collected with informed consent at Platelet Services, BioCity, Pennyfoot Street, NG1 1GF, Nottingham, United Kingdom and at Tan Tock Seng Hospital, Singapore, approved by the institutional review board of Nanyang Technological University (IRB-2019-03-011, IRB-2021-01-037) and Tan Tock Seng Hospital (DSRB 2018/00880, DSRB 2020/01324) in compliance with the Human Biomedical Research Act (Ministry of Health, Singapore), and processed the same day in a Class II biosafety cabinet. Blood was obtained from healthy individuals, both males and females, aged from 21 to 60 years, which represent a broad ethnic group distribution. Importantly, each individual was not under any medications at the time of donation. Furthermore, each biological replicate in this study was from neutrophils isolated from unique, different donors; no donor was used more than once across experiments. Notably, samples were processed within 30 minutes of collection, and all experiments were performed using these freshly isolated neutrophils. In particular, all procedures were performed at room temperature (RT, ∼21 °C) in sterile, Ca^2+^/Mg^2+^-free buffers to minimize inadvertent activation. Citrate-anticoagulated whole blood was centrifuged (15 min, 2,200 rpm, brake off, RT) to remove plasma, then the original volume was restored with DPBS without Ca^2+^/Mg^2+^. Neutrophils were purified by negative selection using the EasySep™ Direct Human Neutrophil Isolation Kit (STEMCELL #19666) with serial magnetic separations employing Isolation Cocktail and RapidSpheres™ (5–10 min each). The clarified fraction containing enriched neutrophils was collected after the third separation, washed twice in PBS (5 min, 1,300 rpm, RT), and processed immediately. Cells were counted by countess automated cell counter and viability assessed by trypan-blue exclusion (typically >95–99%). Representative preparations were verified by morphology and/or CD16/CD66b staining before use.

### 2.4 NETosis quantification by fluorimetry

After neutrophil isolation, cells were resuspended in F12K medium at a concentration of 1.11 × 10^6^ cells/mL. SytoxGreen (Thermo) was added to the cell suspension to a final concentration of 5 μM for the duration of the experiment. A total of 1 × 10^5^ cells were seeded into each well of a 96-well clear bottom black plate that had been previously POLY-L-lysine coated. The plate was then centrifuged for 30 s at 400 g at room temperature to facilitate cell attachment.

Following centrifugation, cells were incubated for 30 min in a humidified 37 °C, 5% CO_2_ incubator to allow for cell attachment to the plate. After attachment, a pre-mixed solution of CaCl_2_ and CXCL8 (10×) in F12K culture medium was added to each well at the specified concentrations. Final nominal CaCl_2_ concentrations range went from 0.125 to 2 mM. Throughout this manuscript, CaCl_2_ concentrations are reported as nominal values, excluding the basal calcium already present in the culture medium. This convention was adopted to ensure reproducibility and comparability across media formulation. Importantly, under our experimental conditions, background calcium did not significantly affect NET formation, as CXCL8 alone did not induce NETosis. Moreover, when CaCl_2_ was prepared in F12 at pH <7.8, a condition that prevents crystal formation, neither CaCl_2_ alone nor its combination with CXCL8 induced NETosis, indicating that microcrystals rather than soluble calcium ions derive NET formation. Inhibitors, if used, were pre-incubated for 30 min in the incubator before stimulation with CaCl_2_/CXCL8.

At time 0, fluorescence was measured using a ClaristarPlus plate reader (Excitation/Emission: 504/523 nm) to obtain baseline fluorescence for normalization purposes. The plate was then incubated for 4 h in the same humidified 37 °C, 5% CO_2_ conditions, and the measure was repeated.

In particular, Relative Fluorescence Units (RFU) at time 0 were subtracted from the RFU at 4 h, and background-subtracted RFU values were plotted for each experimental condition using GraphPad Prism.

### 2.5 NETosis quantification by incucyte

Neutrophils were obtained from buffy coats of citrate–phosphate–dextrose–treated blood from healthy medication-free volunteers, provided by the Transfusion Medicine Service Laboratory, “San Salvatore” Hospital (L’Aquila, Italy). Cells were purified using the MACSxpress® Whole Blood Neutrophil Isolation Kit (Miltenyi Biotech). Neutrophils were resuspended in Ham’s F12K medium and assessed for cell viability, by trypan blue exclusion.

For NETosis assays, neutrophils were resuspended at 1.2 × 10^6^ cells/mL in Ham’s F12K medium containing 40 nM Sytox Green. Fifty μl of cell suspension were seeded onto poly-L-lysine–coated Incucyte® Imagelock 96-well plates and allowed to adhere for 30 min at 37 °C. Stimulations were performed by adding 50 μL of a 4× CaCl_2_ solution in Ham’s F12K followed by 100 μL of 2× stimulants. NETosis was monitored using the Incucyte® S3 Live-Cell Analysis System (Essen BioScience) as described (Gupta et al., 2018). Phase-contrast and green fluorescence images (Excitation/Emission 504/523 nm; exposure 400 ms) were acquired every 30 min for 4 h using a ×20 dry lens objective. Each condition was tested in triplicate.

Image analysis was performed with the Incucyte® ZOOM software. Parameters were optimized to identify green-positive cells undergoing membrane damage: Top-Hat segmentation (radius 30 μm; threshold 4 GCU; edge sensitivity −20), with filters excluding objects <80 μm or >5,000 μm. Mean and integrated intensities <2 were excluded. The same processing definition was applied to all experiments. NETosis was quantified as the average green area per well at defined time points (e.g., 120 min), and data were exported using the Incucyte® Basic Software.

### 2.6 Neutrophil impedance phenotyping

The microfluidic impedance cytometer used for neutrophil profiling was described in previous literature (Petchakup et al., 2019). Treated neutrophils (3 x 10^5^ cells per condition) were retrieved from the 96-well plate after 2 h of incubation and resuspended to a final volume of 1 mL of Ham’s F-12K Medium for impedance measurement. They were loaded into a polydimethylsiloxane (PDMS) chip containing microfabricated electrodes and were hydrodynamically focused into a single stream before passing through a detection region. As each cell crossed the electrodes, it disrupted the alternating current (AC) electric field, producing distinct impedance signals. Prior to the experiment, the device was passivated with 1% bovine serum albumin (BSA, Biowest) in phosphate buffered saline (PBS, Lonza) for 1 h to prevent any non-specific adhesion. The neutrophil sample and sheath were perfused into the device at a flow rate of 70 μL/min and 1,470 μL/min respectively (1:21) using two syringe pumps (PHD ULTRA™, Harvard Apparatus), and the flow was stabilized for 1 min before measurement. Using multi-frequency excitation (0.3 MHz, 1.72 MHz, and 12 MHz), we obtained three key electrical signatures for each neutrophil: (1) nucleus opacity (reflecting nuclear morphology and size), (2) membrane opacity (related to membrane properties such as capacitance), and (3) electrical size (indicating cell -size). By plotting 2D scatter plots of electrical size versus nucleus opacity, we were able to sensitively detect early biophysical changes in neutrophils undergoing NETosis (Petchakup et al., 2022). Each condition was measured for 3 min to record enough events.

### 2.7 Mechanism of action of calcium crystal aggregates

To unravel the signaling pathways leading to NETosis, we used the CXCR1/2 specific antagonist SCH527123 (Sigma-Aldrich) at the concentrations of 0.1, 1, 10, 100 μM, preincubated for 30 min, prior to stimulation. In addition, we also used several known inhibitors of the implicated pathways: Wortmannin, PI3K inhibitor; PP2, protooncogene tyrosine protein kinase Src inhibitor; diphenylene iodonium chloride, NADPH oxidase inhibitor; cytochalasin-B, actin filament elongation inhibitor; CHIR-98014, glycogen synthase kinase 3 (GSK3β) inhibitor; iCRT3, Wnt/β-catenin inhibitor; rapamycin, mammalian target of rapamycin (mTOR) kinase inhibitor; ASN007, extracellular signal-regulated kinases 1/2 (ERK1/2) inhibitor; lonafarnib, Ras inhibitor, and PD988059, mitogen-activated protein kinase kinase (MEK) inhibitor. Inhibitors were tested at concentrations of 0.3, 3, and 30 μM, as also shown in the respective graphs and pre-incubated for 30′ before stimuli. All experiments were performed three times independently. All compounds were purchased from Sigma-Aldrich, except for ASN007, which was obtained from MedChemExpress.

### 2.8 Calcium aggregate analysis by dynamic light scattering (DLS)

We used Litesizer 500 (Anton Paar, Austria) DLS instrument to characterise the nature of the particulate matter formed in the medium following the addition of CaCl_2_. To this end, medium batches prepared with or without CXCL8 were analyzed in terms of aggregate size (nm), polydispersity index (PDI) and transmittance (%). Briefly, following their preparation, all medium batches were kept for 1 h at room temperature and then incubated at 37 °C. All measurements were performed at predefined timepoints (0, 1 h at 25 °C and 4 h at 37 °C). One ml of sample was placed into polystyrene cuvette and inserted into the instrument. The analysis after 4 h of incubation was performed on samples diluted 1:10 (v/v) at analytical temperature of 37 °C.

All experiments were performed three times independently.

### 2.9 Experimental procedures *in vivo*

Male C57BL/6 mice (8–9 weeks old, 18–22 g, Charles River, Calco, Italy) were used to investigate the NETosis *in vivo*. Animals were housed in a controlled environment (temperature 21 °C ± 2 °C and humidity 60% ± 10%) with a 12-h light/dark cycle at the Department of Pharmacy (University of Naples, Italy) with free access to food and water. Animals were acclimatized to laboratory settings for at least 4 days before testing and were used only once throughout the experiments. The daily experimental procedures were always performed at the same time and respected by the light/dark cycle. All procedures in this study were performed following the ARRIVE 2020 guidelines, Italian regulations (D.Lgs. 26/2014) and European Economic Community regulations (EU Directive 2010/63/EU). Mice were treated intraperitoneally with 0.2 mL of saline solution (0.9% NaCl; control group), CXCL8 (50 μg/mL in saline; Giotto Biotech), sublethal dose of LPS (20 mg/kg in saline), CaCl_2_ (10 mM in Ham’s F12K medium), CXCL8 (50 μg/mL) + CaCl2 (10 mM). Following the treatment, mice were anesthetized using 4% isoflurane and then euthanized by cervical dislocation at 4 h and 24 h after stimulus administration. Serum samples were obtained from blood collected through an intracardiac aspiration. Aliquots of serum were stored at −20 °C until they were used for ELISA assay (Cayman Chemical #501620) to evaluate the levels of citrullinated histone H3 (H3Cit).

### 2.10 Statistical analysis

Statistical significance was first determined by one-way or two-way ANOVA followed by Tukey’s, Sidak’s multiple comparisons, or uncorrected Tukey’s or Fisher’s LSD test, as specificed in the figure legends. All analysis were performed using GraphPad Prism10 software.

## 3 Results

### 3.1 CXCL8 potentiates calcium carbonate crystal–induced NETosis *in vitro*

To investigate the role of calcium crystals and their interaction with CXCL8 in mediating NETosis, we developed a protocol for producing calcium carbonate (CaCO_3_) crystals based on the method by Koutsoukos and Kontoyannis (Koutsoukos and Kontoyannis, 1984) (Figure 1A).

We first investigated the effects of varying concentrations of CXCL8, calcium crystals, and their combination on the induction of NETosis using a fluorescent-based NETosis assay with a multi-well plate reader (Figure 1B). Specifically, following neutrophil isolation, cells were incubated with 5 μM SYTOX Green for 30 min, then seeded into a 96-well clear-bottom black plate at a density of 10^5^ cells per well, and treated with varying concentrations of CXCL8 and/or crystal suspensions in F12K culture medium for 4 h.

Although CXCL8 alone (1–1,000 ng/mL) failed to significantly induce NETosis under our experimental conditions, it markedly and robustly enhanced crystal-mediated NET formation (Figure 1B). Notably, when neutrophils were treated with 0.25, 0.5, 1, and 2 mM calcium crystal suspensions, the addition of CXCL8 at several concentrations triggered a highly significant increase in NETosis (p < 0.0001), compared to calcium crystals alone. The effect of CXCL8 on NETosis initiates at lower concentrations (1–10 ng/mL) and becomes increasingly robust at higher doses. Although concentrations exceeding 10 ng/mL are considered supra-physiological, we utilized 100 ng/mL of CXCL8 and 1 mM of calcium crystals for the remainder of the experiments. These specific conditions were selected to ensure experimental reproducibility and to avoid potential masking effects driven by calcium crystals.

To standardize the protocol for calcium crystal–induced NETosis, we optimized the conditions for crystal formation before their addition to isolated human neutrophils. Given the time-dependent nature of crystal precipitation, we evaluated the impact of various pre-incubation times of 10 mM CaCl_2_ solutions in culture media, subsequently diluted 1:10 to reach a final concentration of 1 mM at cell contact. In particular, CaCl_2_ solutions were pre-incubated for 5, 15, 30, 45, 60, 90, or 120 min, then added to neutrophils in the presence or absence of CXCL8 (100 ng/mL), followed by a 4-h incubation (Supplementary Figure S1). NETosis was then quantified (Supplementary Figure S1). Our results revealed a time-dependent increase in NETosis, with longer pre-incubation times of CaCl_2_ correlating with enhanced NET formation. Notably, the 120-min pre-incubation yielded highly consistent results, with one of the most robust and statistically significant increases in NETosis in the “crystals and CXCL8” condition compared to crystals alone (Supplementary Figure S1). Therefore, this time-point was adopted as the standard condition throughout the study. Notably, this enhancement is likely attributable to increased calcium crystal formation over time, as confirmed by dynamic light scattering (DLS) analysis (Supplementary Figure S2). DLS measurements showed a progressive increase in calcium aggregate size, with the average diameter increasing from <550 nm at the initial time-point to approximately 1,100 nm after 1 h, eventually plateauing at around 1,250 nm after 4 h of incubation at 37 °C (Table 1; Supplementary Figure S2). To further investigate these findings, we employed the Incucyte® SYTOX Green Dye and Incucyte® ZOOM live-cell imaging system, a live, well-standardized and automated technology for NET quantification (Gupta et al., 2018). In particular, isolated human neutrophils were treated with 100 ng/mL CXCL8, calcium crystals generated (1 mM CaCl2 media suspension), or a combination of both. NETosis was assessed by live imaging at 2- and 4-h post-treatment. Consistent with prior observations, calcium crystals triggered NETosis, and this effect was significantly potentiated at both time points when combined with CXCL8 (Figures 1C,D).

**TABLE 1:** Dimensional analysis, polydispersity index and transmittance of Ham’s F12K medium with CaCl_2_ or CaCl_2_ + CXCL8.

Condition	Time (h)	Size (nm)	PDI	Transmittance (%)
Ham’s F12K + CaCl_2_	0	507.1	0.221	44.1
Ham’s F12K + CaCl_2_ + CXCL8		527.3	0.212	52.1
Ham’s F12K + CaCl_2_	1	966.3	0.21	27.0
Ham’s F12K + CaCl_2_ + CXCL8		1,004.0	0.23	38.5
Ham’s F12K + CaCl_2_	4	1,200.9	0.310	51.0^a^
Ham’s F12K + CaCl_2_ + CXCL8		1,247.7	0.387	52.1^a^

^a^ Analysis performed on medium diluted 1:10 that justifies the increase in transmittance; PDI, polydispersity index.

### 3.2 CXCL8 amplifies NETosis across crystal types and culture conditions

To determine whether NETosis was induced by the increase in calcium molarity in solution rather than insoluble CaCO_3_ crystals, neutrophils were treated with 1 mM CaCl_2_, in F12K media at pH < 7.8. At this lower pH calcium remains mainly soluble as Ca^2+^ ions, preventing crystal precipitation, and no significant NETosis was observed either with CaCl_2_ alone or in combination with 100 ng/mL CXCL8 (Supplementary Figure S3). These findings strongly suggest that the potentiating effect of CXCL8 on crystal-mediated NETosis is dependent on the physical presence of calcium crystals rather than on soluble calcium in the medium. Moreover, to determine whether the ability of CXCL8 to enhance crystal-mediated NETosis is maintained also with other types of calcium crystals, we included in our NET assays the pre-made insoluble calcium pyrophosphate dihydrate (CPPD) crystals (10 μg/mL) (Supplementary Figure S4), which are known to cause acute synovial inflammation in pseudogout (Liu-Bryan and Lioté, 2005). Consistent with our earlier findings, CXCL8 further enhanced NET formation induced by CPPD crystals compared to stimulation with crystals alone (Supplementary Figure S5), reinforcing the concept that CXCL8 can amplify crystal-driven NETosis independent of calcium crystal composition.

**FIGURE 2:**
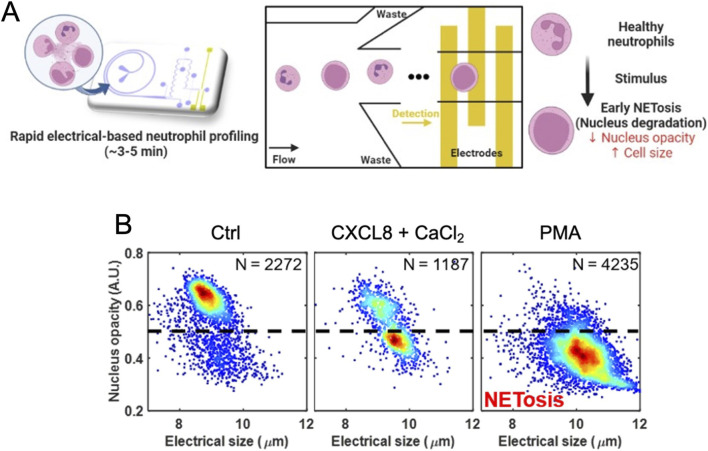
Schematic illustration of the microfluidic platform (NoC) designed for label-free, single-neutrophil impedance phenotyping. **(A)** – Neutrophils and sheath fluid were introduced into the device at flow rates of 70 μL/min and 1,470 μL/min respectively (1:21 ratio). Electrical signatures of individual cells were measured at three frequencies (0.3 MHz, 1.72 MHz, and 12 MHz) to determine nucleus opacity, membrane opacity, and electrical size. Each experimental condition was measured for 3 min to ensure sufficient event recording. The hallmarks of NETotic cells include a reduction in nuclear opacity and an increase in overall cell size. **(B)** – 2D density impedance scatter plots of nucleus properties versus electrical size for untreated (Ctrl), 100 ng/mL CXCL8 with 1 mM CaCl_2_ and PMA (100 nM) treated neutrophils after 2 h. Dotted line indicates nucleus opacity (<0.5) threshold for NETosis events.

Importantly, to assess whether the effect on NETosis was reproducible in other media, CaCl_2_ suspensions were prepared in RPMI1640, another nutrient-rich culture medium. The results were similar to those obtained with F12K, showing that adding CaCl_2_ to RPMI1640 medium induced crystal formation and NETosis, an effect further potentiated by CXCL8 (Supplementary Figure S6). Finally, to provide mechanistic insight beyond conventional fluorescence assays and to capture the earliest cellular events of NET formation, we employed an innovative real-time, label-free impedance profiling approach (Figure 2A). This technology enables highly sensitive quantification of early NETosis-associated changes in nuclear opacity and cell size at the single-cell level, events that precede membrane permeabilization and SYTOX uptake (Hoppenbrouwers et al., 2017; Petchakup et al., 2019). By applying this complementary method, we were able to detect the initial biophysical commitments of neutrophils to NETosis induced by the combination of CXCL8 and calcium crystals. Neutrophils treated with Phorbol Myristate Acetate (PMA), a potent NETosis inducer used as a positive control, exhibited a marked increase in cell size and a reduction in nucleus opacity relative to untreated controls (Figure 2B). These changes are consistent with chromatin decondensation and swelling, hallmark features of NETosis (Neubert et al., 2018), and closely matched impedance simulations showing that increased nuclear size corresponds to decreased nuclear opacity (a ratio of current response at 12 MHz–0.3 MHz) (Petchakup et al., 2019). Strikingly, neutrophils stimulated with the combination of CXCL8 and calcium crystals exhibited impedance profiles comparable to those induced by PMA, indicating that the CXCL8–crystal synergy triggers biophysical changes reminiscent of those induced by classical NETosis inducers, despite acting through different upstream mechanisms. Specifically, this combined treatment revealed two distinct neutrophil populations: one displaying high nuclear opacity, typical of resting cells, and another characterized by low nuclear opacity, indicative of early NETosis events.

### 3.3 CXCL8 potentiates calcium crystal–induced NETosis via Src, PI3K and ERK1/2 pathways

**FIGURE 3:**
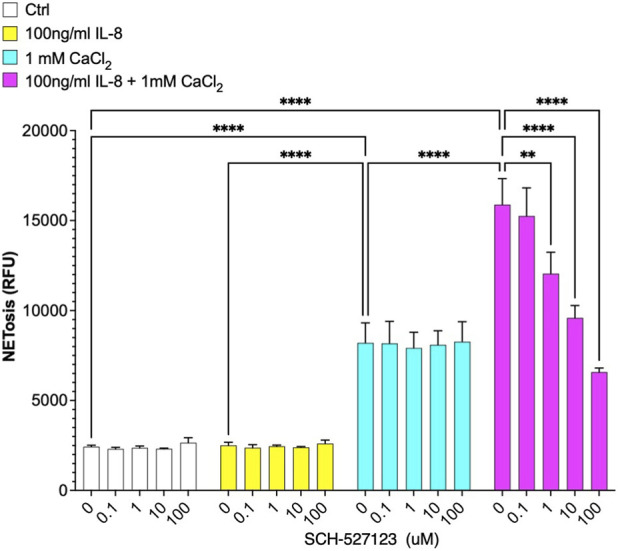
Mechanism of CaCl_2_ + CXCL8-induced NETosis. The non-competitive CXCR1/CXCR2 antagonist SCH527123 blocks the CXCL8-dependent component of NETosis induced by the combination of 1 mM CaCl_2_ and 100 ng/mL CXCL8. SCH527123 was pre-incubated with neutrophils for 30 min prior to stimulation. Statistical analysis was performed using two-way ANOVA followed by uncorrected Fisher’s LSD multiple comparisons test.

To investigate the enhancing effects of CXCL8 signaling on calcium crystal mediated NETosis we utilized SCH-527123, a selective CXCR1/CXCR2 receptor antagonist (Gonsiorek et al., 2007). In particular, different concentrations of the SCH-527123 compound were preincubated 30 min before a 4-h stimulation with either CXCL8, calcium crystals or their combinations, as shown in Figure 3. Strikingly, inhibition of these receptors led to a significant, dose-dependent reduction in NETosis in neutrophils treated with the combination of CaCl_2_ and CXCL8, bringing NETosis levels down to those observed with calcium crystals alone (Figure 3). Notably, SCH-527123 had no effect on NETosis induced by CaCl2 alone (Figure 3), confirming the specific involvement of CXCR1 and CXCR2 activation by CXCL8 in potentiating the crystal-induced NET formation.

**FIGURE 4:**
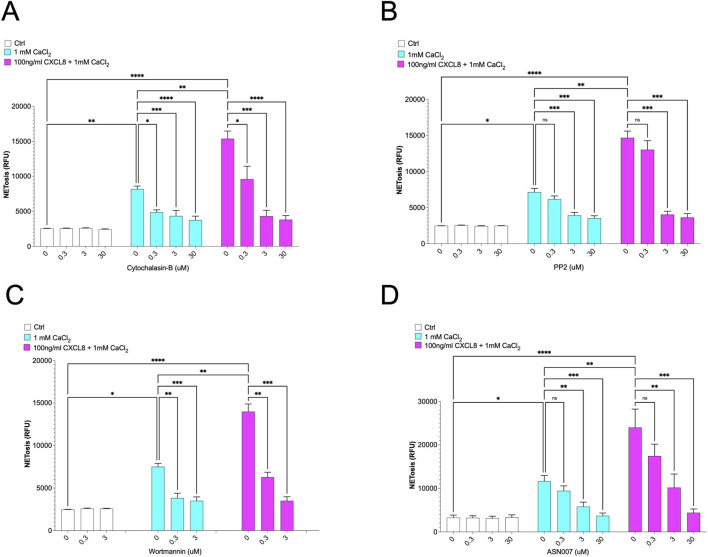
Suppression of CaCl_2_ and CaCl_2_ + CXCL8-induced NETosis by pharmacological inhibitors. **(A)** – Cytochalasin B (inhibitor of actin polymerization), **(B)** – PP2 (inhibitor of Src-family kinases), **(C)** – Wortmannin (inhibitor of PI3K), and **(D)** – ASN007 (inhibitor of ERK1/2 pathways) all reduce NETosis induced by 1 mM CaCl_2_ alone or in combination with 100 ng/mL CXCL8. Inhibitors, at indicated concentrations, were pre-incubated with neutrophils for 30 min prior to stimulation. Statistical analysis was performed using two-way ANOVA followed by uncorrected Fisher’s LSD multiple comparisons test.

Notably, NET formation is also closely associated with cytoskeletal rearrangements, including actin polymerization and integrin clustering, which are essential for neutrophil spreading and degranulation during vital NETosis (O’Brien and Reichner, 2016; Thiam et al., 2020; Sprenkeler et al., 2022). Consistent with this notion, our findings demonstrate that NETosis in our *in vitro* model requires active cell movement, as shown by the complete inhibition of NET formation following treatment with cytochalasin B, an inhibitor of actin polymerization (Cooper, 1987) (Figure 4A). Finally, we demonstrated that inhibitors of Src (PP2), PI3K (wortmannin), and ERK1/2 (ASN007) pathways, each implicated in the regulation of cytoskeletal rearrangements, effectively blocked NET formation in a dose-dependent manner. These findings highlight the essential role of these pathways in NETosis induced by both calcium crystals alone and in combination with CXCL8 (Figures 4B–D). Importantly, we ruled out the involvement of several other signaling pathways commonly associated with NETosis by using increasing concentrations of specific inhibitors targeting NADPH oxidase (DPIC), GSK3 (CHIR-98014), Wnt/β-catenin (iCRT3), mTOR (rapamycin), RAS (lonafarnib), and MEK (PD98059) (Supplementary Figure S7A–F).

### 3.4 CXCL8 enhances calcium crystals mediated NETosis *in vivo*

**FIGURE 5:**
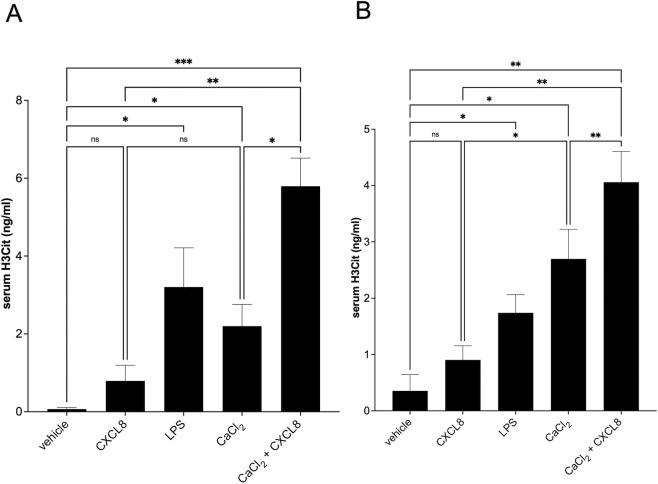
*In vivo* induction of NETosis by CaCl_2_ and CaCl_2_ + CXCL8, measured by serum levels of citrullinated histone H3 (H3Cit). Intraperitoneal administration of 10 mM CaCl_2_ or the combination of 10 mM CaCl_2_ and 50 μg/mL CXCL8 induced NETosis in healthy C57BL/6 mice, as indicated by elevated serum levels of citrullinated histone H3 (H3Cit), compared to control (saline), CXCL8 (50 μg/mL), or LPS (20 mg/kg) treatments. **(A)** – Serum levels of H3Cit at 4 h **(B)** – Serum levels of H3Cit at 24 h. Statistical analysis was performed using one-way ANOVA followed by uncorrected Fisher’s LSD multiple comparisons test.

To investigate the impact of CXCL8 in potentiating calcium crystal-mediated NETosis *in vivo*, we intraperitoneally injected CXCL8 (50 μg/mL), calcium carbonate crystals from a suspension of 10 mM CaCl2, and their combination into male C57BL/6 mice. These treatments were compared to the LPS-induced sepsis model (Tanaka et al., 2014). Induced NETosis was detected as an increase in serum levels of citrullinated histone H3 (H3Cit) (Masuda et al., 2016). Consistent with the *in vitro* studies, CXCL8 alone did not significantly induce NETosis, whereas treatment with CaCl2 suspension alone reported a significant increase of H3Cit serum levels both at 4 (Figure 5A) and 24 h post-treatment (Figure 5B). Once again, the combination of calcium crystals and CXCL8 enhanced the induction of NETosis compared to crystals alone, both at 4 h and 24 h post-treatment (Figures 5A,B). As expected, LPS induced robust NETosis *in vivo*, validating the sensitivity of our model.

## 4 Discussion

**FIGURE 6:**
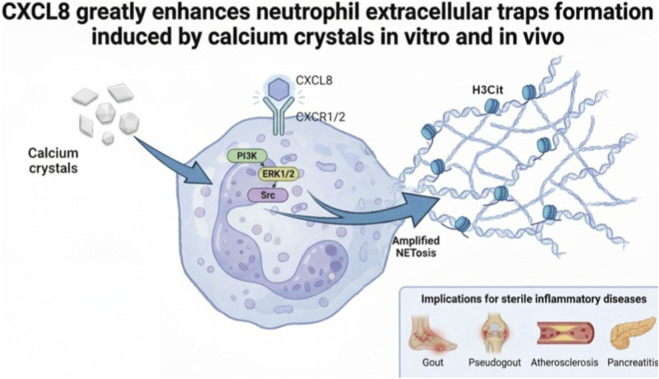
Schematic representation of CXCL8-induced amplification of Crystal-mediated Netosis. Calcium crystals induce NET formation, which is markedly enhanced by CXCL8-mediated activation of CXCR1/2. The figure was created by using FigureLabs, an AI scientific illustration tool.

Our findings provide a novel mechanistic resolution to the long-standing controversy regarding the ability of CXCL8 to induce NETosis (Figure 6). By introducing a biologically plausible “two-signal” model, we demonstrate that CXCL8 is not a universal or autonomous inducer of NET formation, but rather functions as a context-dependent amplifier. This study offers one of the first clear demonstrations that CXCL8 cooperates with calcium-based crystalline DAMPs, specifically CaCO_3_ and CPPD, to markedly enhance NETosis. By explicitly positioning CXCL8-mediated NET amplification as a crystal-dependent process, our work reconciles the apparent discrepancies in previous literature and highlights the clinical significance of this crosstalk in sterile inflammatory pathologies such as gout, atherosclerosis, and pancreatitis. We observed that the extent of NETosis correlates with both size and quantity of calcium aggregates interacting with neutrophils, regardless of the crystal’s chemical nature, as CaCO_3_ and preformed CPPD crystals exhibited similar effects. Notably, these combinations promote NETosis via Src, PI3K, and ERK1/2 signalling pathways, which are all well-known to play key roles in cytoskeletal rearrangements, particularly in immune cells like neutrophils. Consistent with this context-dependent model, we observed that the pro-NETosis effect of CXCL8 becomes detectable at relatively low concentrations (1-10 ng/mL) and increases progressively at higher doses. Although concentrations above 10 ng/mL are generally considered supra-physiological, we used 100 ng/mL CXCL8 in combination with the calcium crystals to ensure a response that was both robustly analyzable and comparable across different conditions.

Although some studies have reported that CXCL8 can directly promote NETosis *in vitro*, this effect appears to be highly context-dependent and is not consistently observed across different experimental systems (Hoppenbrouwers et al., 2017). In line with prior reports showing context-dependent effects (Hoppenbrouwers et al., 2017; Teijeira et al., 2021), CXCL8 alone elicited only a weak and non-significant trend, towards NETosis in our experimental conditions, whereas it significantly and robustly enhanced calcium crystal-mediated NETosis. Strikingly, this promoting effect was also reproduced *in vivo*. Specifically, C57BL/6 mice treated intraperitoneally with both calcium crystal suspension and CXCL8 exhibited significantly elevated serum levels of citrullinated histone H3 (H3Cit), a well-established specific marker of NETosis, compared to those receiving calcium crystal suspension alone. These findings highlight the potent proNETotic effect of the combination “calcium crystals and CXCL8” and strongly suggest that this mechanism may play a substantial role in the progression of diseases characterized by pathological crystal depositions, echoing observations from other models of sterile inflammation (Warnatsch et al., 2015) and establishing a mechanistic and functional link between CXCL8–crystal synergy and systemic NETosis.

Notably, the molecular mechanisms involved in NETosis may vary depending on the stimulus. For instance, while PMA, A23187, and mineral particles require the formation of reactive oxygen species (ROS), nigericin treatment induces NETosis without triggering ROS production (Kenny et al., 2017; Peng et al., 2017; Wang et al., 2019). Although the exact mechanism by which calcium crystals induce NETosis remains to be fully elucidated, our findings are consistent with those of Tan et al. (2023) who demonstrated that urate crystals promote NETosis via a NADPH oxidase-independent pathway. Furthermore, it has been reported that calcium crystals are initially recognized by type I transmembrane receptors on neutrophils, such as CD32 and CD11b, which in turn trigger a robust tyrosine kinase signaling cascade. This cascade involves Src-family kinases like Lyn, as well as other key signaling molecules including conventional protein kinase C (PKC), Syk, Tec, and PI3Ks. Strikingly, in our model, inhibition of NADPH oxidase, GSK3, Wnt/β-catenin, mTOR, RAS, and MEK pathways did not impair NETosis mediated by calcium crystals or by the combination “calcium crystals and CXCL8”, supporting the notion of a ROS-independent mechanism of NETs formation. These results suggest that the observed NETosis may represent a form compatible with vital NETosis, rather than a lytic one.

Remarkably, CXCL8 significantly enhanced calcium crystal-induced NETosis, resulting in a greater NET response compared to crystals alone suggesting that this phenomenon could have substantial relevance in pathologies such as atherosclerosis or pancreatitis that are characterized by crystal depositions and cartilage degradation in osteoarthritis associated with CPPD disease (Leppkes et al., 2016; Ejaz et al., 2020; Pascart et al., 2024). Supporting this concept, NETs have been found in atherosclerotic plaques and blood clots in both humans and mice. They activate immune cells such as endothelial cells, antigen-presenting cells, and platelets, which can trigger inflammation and promote the development of atherosclerosis and arterial thrombosis (Borissoff et al., 2013; Döring et al., 2017). Cholesterol and calcium phosphate crystals, commonly present in atherosclerotic plaques, can strongly trigger NETosis. This, in turn, worsens inflammation and promotes blood clot formation by activating platelets and boosting the immune response (Ejaz et al., 2020). Notably, elevated levels of CXCL8 have even been found in the blood of patients with atherosclerotic plaques, supporting its role in the inflammation that drives plaque formation and progression (An et al., 2019).

Importantly, our protocol for generating calcium aggregates demonstrated that a pH near 8 significantly promotes calcium crystal formation. Pancreatic juice, unlike serum, has a slightly alkaline pH and is supersaturated with calcium and bicarbonate ions, which physiologically neutralize acidic gastric contents after meal (Gerolami et al., 1989). This unique environment may therefore favor the precipitation of calcium as CaCO_3_ crystals within the pancreatic ducts, potentially triggering NETosis. In type 2 autoimmune pancreatitis, a disease often affecting younger patients (Kamisawa et al., 2013), neutrophils are the predominant inflammatory cells infiltrating the pancreas. Our findings on the potentiating effect of CXCL8 on calcium crystal-induced NET formation suggest important therapeutic implications. Specifically, CXCL8 antagonists could reduce excessive NETosis and subsequent duct obstruction, offering a promising strategy to manage pancreatitis. Importantly, *in vivo* inhibition of CXCR2 in murine models of acute and chronic pancreatitis reduced neutrophil recruitment and ameliorated pancreatic injury, supporting the therapeutic potential of targeting the IL-8/CXCR2 axis in crystal-prone ductal environments (Steele et al., 2015).

To further validate the involvement of CXCL8–CXCR1/2 signaling in crystal-induced NETosis, we employed the dual CXCR1/2 antagonist SCH-527123, a well-characterized orthosteric inhibitor frequently used as a reference compound in neutrophil studies. SCH effectively blocked CXCL8-enhanced NET formation, confirming the contribution of CXCR1/2 engagement to this process. Interestingly, the CXCR1/2 allosteric inhibitor Ladarixin, recently shown to selectively impair neutrophil extravasation without affecting adhesion or chemotaxis (Napoli et al., 2025), was not found to inhibit NETosis under the conditions tested, likely reflecting the distinct signaling bias of allosteric versus orthosteric CXCR1/2 inhibitors (data not shown). Acting as a non-competitive modulator at a conserved transmembrane site (Bertini et al., 2004; Bertini et al., 2012), Ladarixin preferentially dampens Gαi3-dependent CXCR2 signaling (Rizo-Téllez and Filep, 2025), whereas orthosteric antagonists such as SCH-527123 exert broader inhibition of CXCL8-driven receptor activation. Further studies are warranted to determine whether these mechanistic differences underlie the divergent effects observed on crystal-induced NETosis.

Furthermore, since microcrystals trigger increased release of CXCL8 during acute gout or pseudogout attacks (Hachicha et al., 1995; Scanu et al., 2021), our results could provide insight into the underlying pathological processes involved in the acute phase of gout or pseudogout attacks. Consistently, in a rabbit model that reproduces acute gout flares by intra-articular injection of MSU crystals, neutralization of IL-8 attenuated arthritis, reducing neutrophil influx and joint swelling (Nishimura et al., 1997; Busso and So, 2010). Specifically, in crystal-associated diseases such as gout, pseudogout, atherosclerosis, and pancreatitis, neutrophil activation and NET formation play a dual role. While contributing to tissue damage (Ma and Steiger, 2024), they may also exert regulatory effects by trapping inflammatory mediators, including chemokines such as CXCL8, thereby limiting their diffusion and reducing neutrophil chemotaxis, as previously reported (Schauer et al., 2014; Maueröder et al., 2015). Building on this framework, our findings show that CXCL8 potentiates crystal-induced NET formation. Therefore, sequestration of CXCL8 within NET structures could limit not only neutrophil recruitment—through the reduced chemotactic gradients described in the literature—but also the CXCL8-mediated amplification of NET formation that we observe in this study. By dampening this positive feedback loop between CXCL8 signaling and crystal-driven neutrophil activation, this mechanism may help explain the rapid resolution and self-limited nature of acute inflammatory flares observed in crystal-associated diseases such as gout (Schauer et al., 2014). These findings also suggest potential therapeutic implications. Our data indicate that pharmacological inhibition of CXCR1/2 signaling may represent a rational strategy to limit excessive NET formation and the resulting tissue damage in crystal-driven inflammatory disorders. Current treatments for conditions such as gout, pseudogout, or crystal-associated pancreatitis rely largely on broad anti-inflammatory agents, including corticosteroids and non-steroidal anti-inflammatory drugs, which suppress inflammatory responses without specifically targeting neutrophil activation pathways (Cowley and McCarthy, 2023; Amodio et al., 2025). In contrast, CXCR1/2 antagonism could simultaneously reduce neutrophil recruitment by limiting CXCL8-driven chemotaxis and prevent amplification of crystal-induced NET formation. Moreover, since NETs can promote platelet activation and thrombosis (Xu et al., 2022), selective targeting of this pathway may also help mitigate thrombo-inflammatory complications associated with crystal deposition. Taken together, our findings suggest that pharmacological agents targeting the CXCL8–CXCR1/2 pathway, could potentially be explored as prophylactic or therapeutic strategies to prevent or attenuate recurrent inflammatory flares in patients predisposed to crystal-mediated sterile NETosis-pathologies.

## 5 Conclusion

This study demonstrates for the first time that CXCL8 signalling acts as a potent amplifier of NET formation rather than as a primary trigger of NETosis. In particular, we established a highly reproducible in vitro model in which the amplifying effect of CXCL8 is robust and quantitatively measurable. This experimental platform provides a powerful tool to dissect the specific contribution of CXCR1/2 signalling to NET formation and to potentially uncover novel pharmacological targets relevant to crystal-associated inflammatory diseases such as pancreatitis. Moreover, we demonstrated that calcium-based microcrystals, including CaCO3 and CPPD, provided a critical priming signal for NETosis through activation of Src, PI3K, and ERK1/2 signaling pathways, thereby creating a permissive signalling environment that enabled CXCL8-driven amplification of NET formation via CXCR1/2 engagement. Notably, NET formation occurs independently of NADPH oxidase activity, supporting the concept that this process represents a ROS-independent, vital-like form of NETosis rather than classical lytic NET formation. This cooperative mechanism is consistently observed both in vitro and in vivo, underscoring its physiological and pathological relevance. By identifying a previously unrecognised crosstalk between chemokine signalling and crystas-mediated NET formation, our findings reconcile previously conflicting reports on CXCL8-induced NETosis and establish a unifying mechanistic framework for excessive NET formation in sterile inflammatory diseases characterised by crystal deposition, including gout, pseudogout, atherosclerosis, and pancreatitis. Importantly, these findings suggest that patients with crystal-driven inflammatory disorders and elevated CXCL8 levels may be especially susceptible to amplified NET-mediated tissue injury and downstream inflammatory complications, thereby strengthening the therapeutic rationale for pharmacologically targeting the CXCL8/CXCR1/2 axis in crystal-associated diseases.

## Data Availability

The original contributions presented in the study are included in the article/Supplementary Material, further inquiries can be directed to the corresponding authors.
