# Supplementary figures and images for: CXCL8 greatly enhances neutrophil extracellular traps formation induced by calcium crystals in vitro and in vivo
Source: Front Pharmacol. 2026 Apr 20;17:1794524. doi: 10.3389/fphar.2026.1794524 (PMC13136156; doi:10.3389/fphar.2026.1794524)

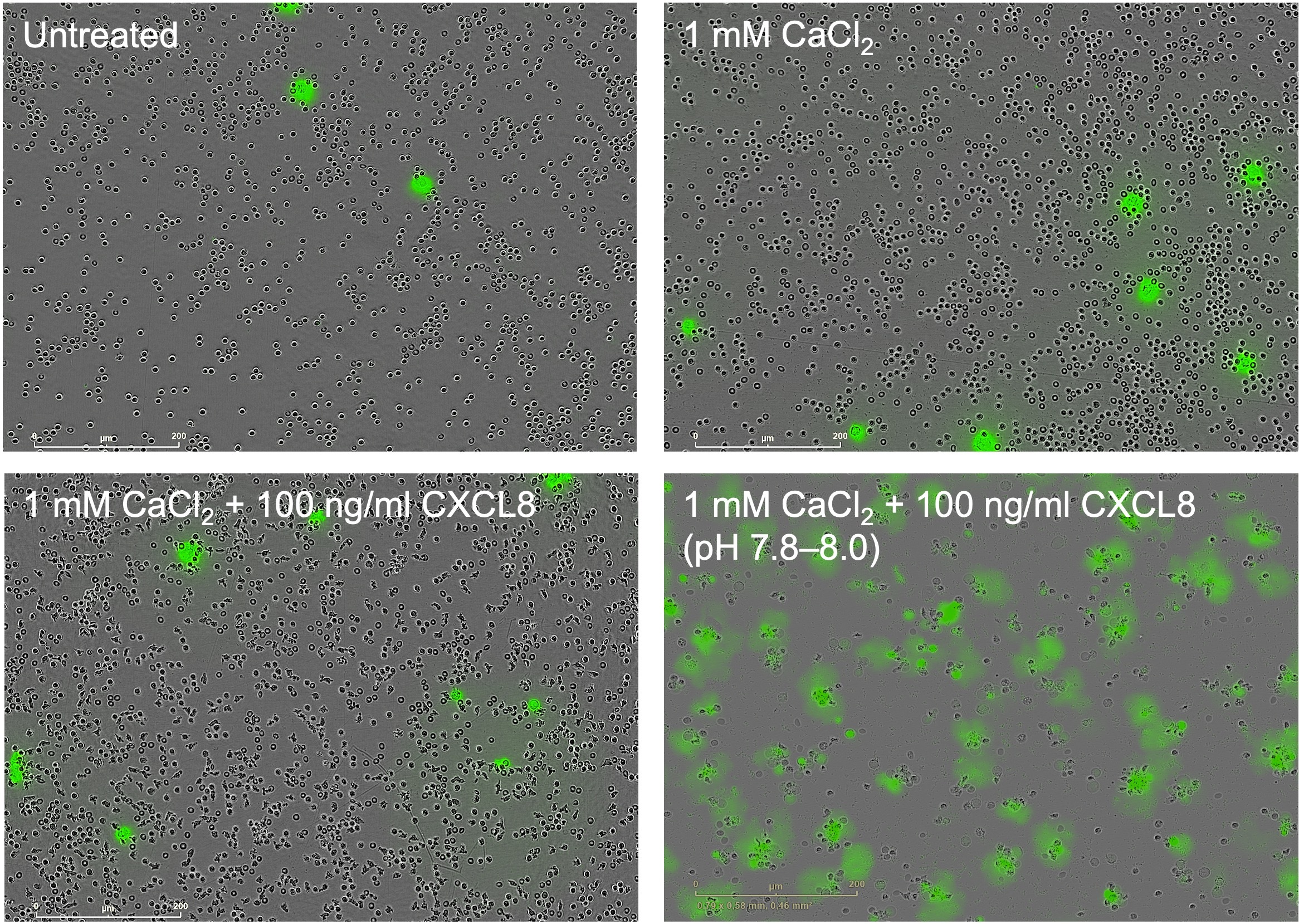

Supplement: Supplementary file 1 [file Image3.jpeg]

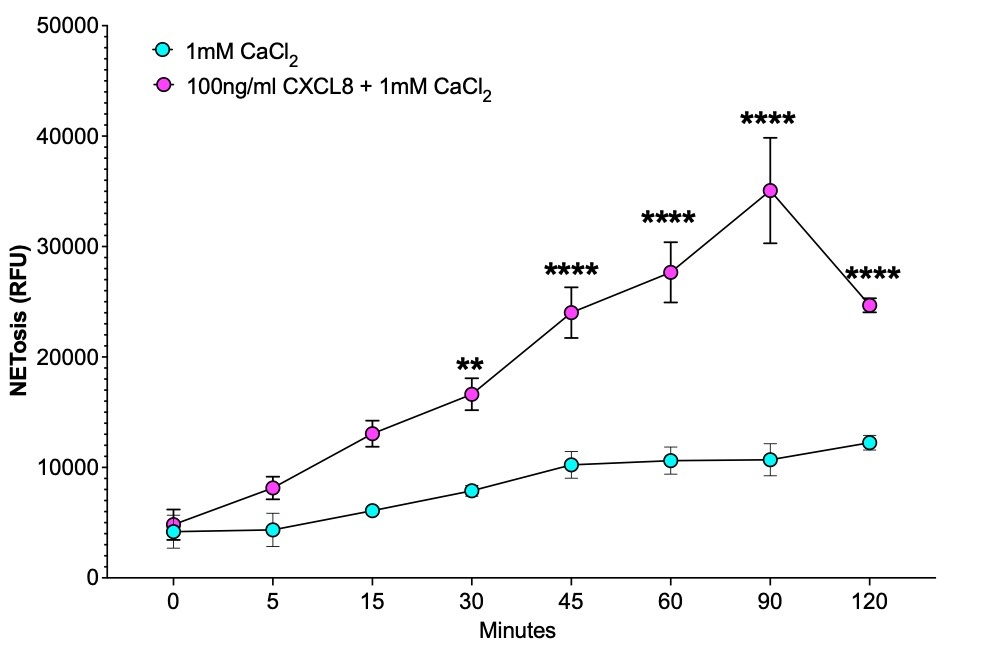

Supplement: Supplementary file 3 [file Image1.jpeg]

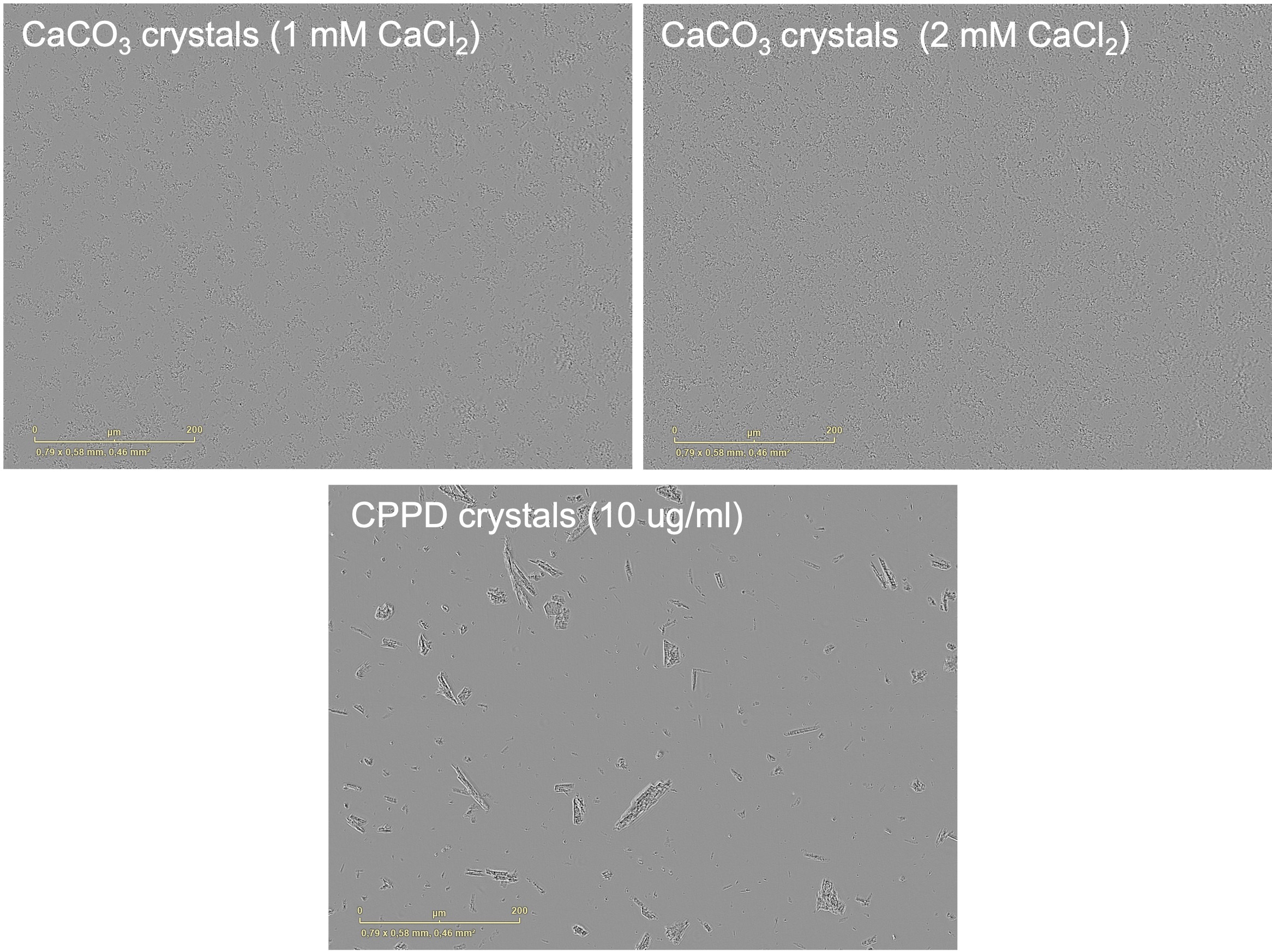

Supplement: Supplementary file 4 [file Image4.jpeg]

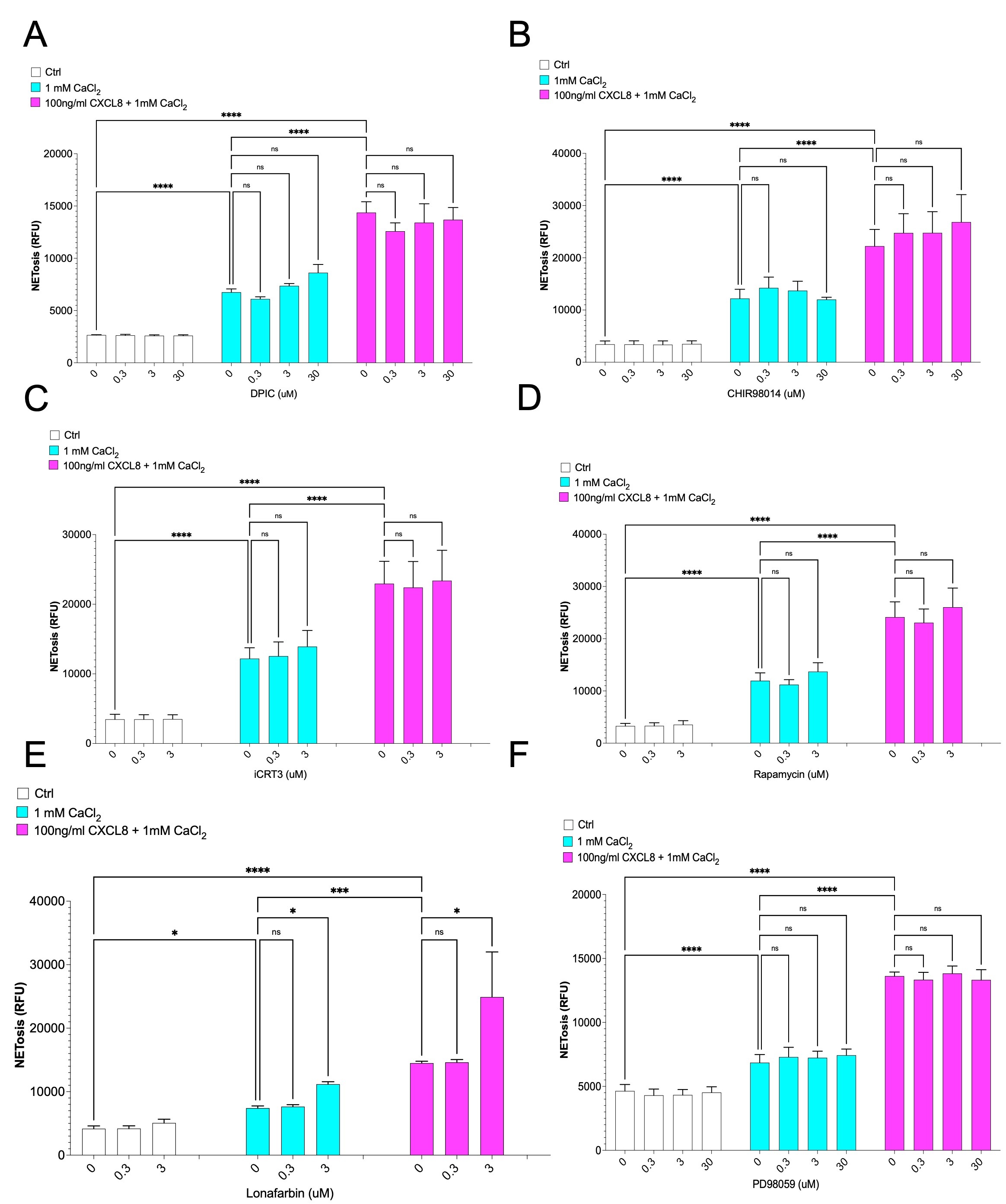

Supplement: Supplementary file 5 [file Image7.jpeg]

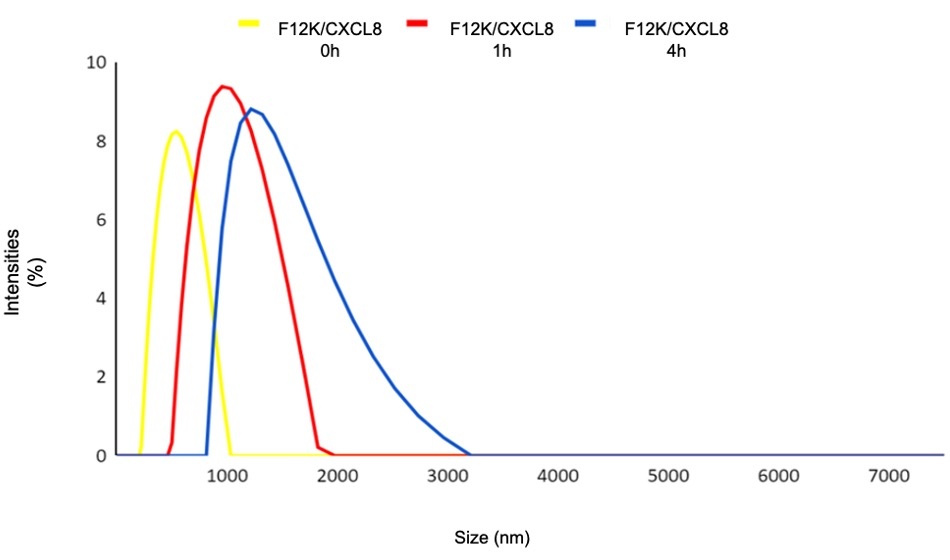

Supplement: Supplementary file 6 [file Image2.jpeg]

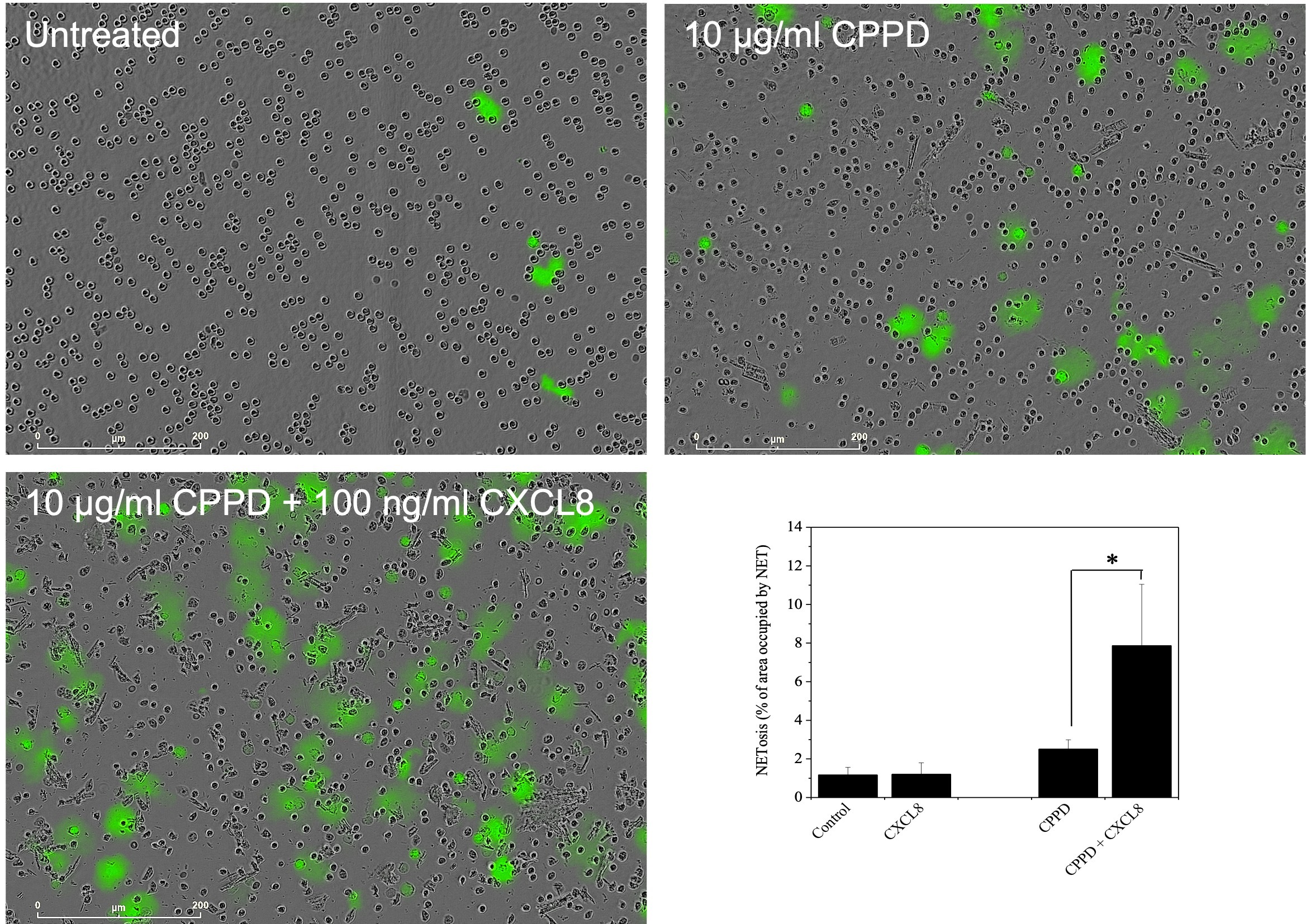

Supplement: Supplementary file 7 [file Image5.jpeg]

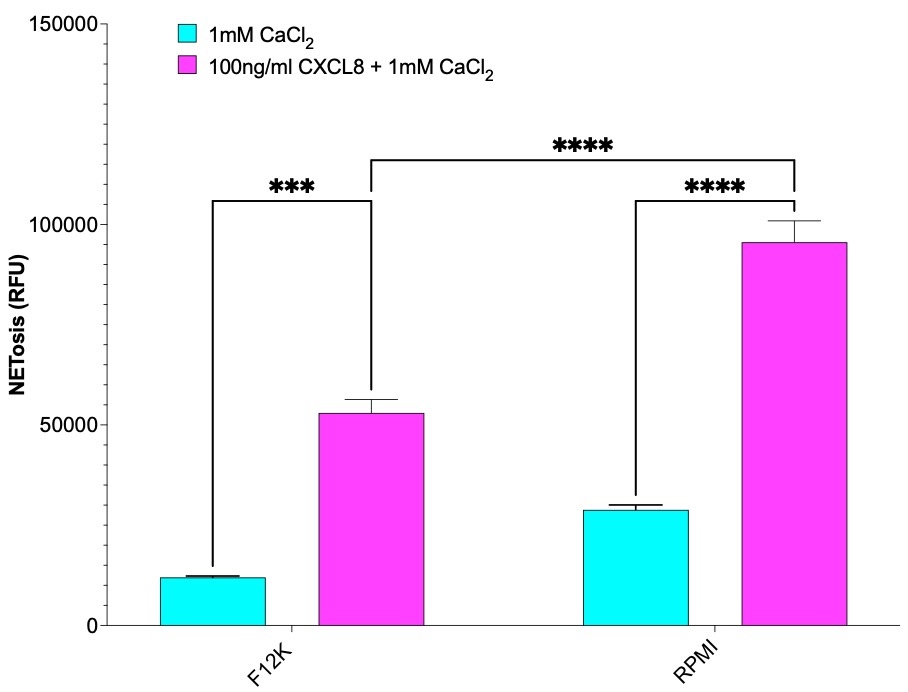

Supplement: Supplementary file 8 [file Image6.jpeg]
