# Supplementary material for: CXCL8 greatly enhances neutrophil extracellular traps formation induced by calcium crystals in vitro and in vivo
Source: Front Pharmacol. 2026 Apr 20;17:1794524. doi: 10.3389/fphar.2026.1794524 (PMC13136156; doi:10.3389/fphar.2026.1794524)
Supplement: Supplementary file 2 [file Supplementaryfile1.docx]

Supplementary Material

**Supplementary figures**

**Figure S1**. **NETosis induced by calcium crystals depends on the incubation time of the 10 mM CaCl₂ pre-mix and the choice of preparation medium**. A 10 mM CaCl₂ pre-mix was prepared in F12K medium, allowing calcium crystals to form, and was subsequently shaken for varying durations before being added to neutrophils in a multiwell plate, either alone or in combination with 100 ng/mL CXCL8 as indicated in graph. After 4 hours, fluorescence was measured using a multiplate reader, revealing that one of the most robust and consistent increases in NETosis occurred at 120 minutes. Statistical analysis: two-way ANOVA followed by Šídák's multiple comparisons test.

**Figure S2**. **Characterization of CaCl₂ aggregates in Ham’s F12K medium with CXCL8 by dynamic light scattering (DLS).** CaCl_2_ aggregates were prepared in Ham’s F12K medium in presence of 100 ng/mL CXCL8 and kept in agitation for 1 hour. All measurements were performed at predefined timepoints (0, 1 hour at 25°C and 4 hours at 37°C). The analysis after 4 hours of incubation was performed on samples diluted 1:10 (v/v) at analytical temperature of 37 °C. All experiments were performed three times independently.

**Figure S3. CXCL8 does not induce NET formation in F12K medium containing 1 mM CaCl₂ in the absence of CaCO₃ crystals**. Neutrophils were incubated in non-alkalinized F12K medium (pH < 7.8) supplemented with 1 mM CaCl₂. Under these conditions, calcium carbonate crystals did not form, and NET formation was not induced, even in the presence of 100 ng/mL CXCL8. In contrast, under our alkaline conditions (pH 7.8–8.0) with 1 mM CaCl₂ (calcium carbonate crystals are formed) and 100 ng/mL CXCL8, robust NET formation was observed. Extracellular DNA was visualized using Sytox Green staining and monitored with the Incucyte ZOOM Analyzer. As a positive control for NETosis, we used neutrophils incubated in alkalinized F12K medium (pH 7.8–8.0).

**Figure S4**. **Calcium crystals and calcium pyrophosphate (CPPD) crystals deposited in F12K medium containing different concentrations of CaCl_2_.** Calcium crystals were formed as described in Materials and methods and Figure 1A, in a pre-mixed solution of 10 mM alkalinized F12K medium. Aliquots of this suspension were then diluted in F12K medium, in order to reach a nominal calcium concentration of 1 and 2 mM. The amount of Calcium crystals was proportional to the nominal concentration of calcium present in the F12K medium. Preformed calcium pyrophosphate dihydrate (CPPD) crystals were weighed and added to the F12K medium in the amount of 10 µg/mL. All images were acquired at 20x magnification with Incucyte ZOOM Analyzer.

**Figure S5**. **CXCL8 enhances NET formation induced by preformed CPPD crystals in F12K medium.** Isolated neutrophils were incubated with 10 µg/mL CPPD crystals in F12K medium in the presence or absence of 100 ng/mL CXCL8. NET formation was visualised using Sytox Green and quantified with the IncuCyte ZOOM Analyser, as shown in the graph. Statistical analysis was performed using ANOVA followed by Tukey’s HSD *post hoc* test.

**Figure S6**. **Comparison between RPMI1640 and F12 on NET formation mediated by crystals in the presence or absence of CXCL8**. Calcium crystals were prepared either in RPMI1640 or in the nutrient-rich F12 medium, and the resulting pre-mix (10 mM CaCl_2_) was added to neutrophils in the presence or absence of 100 ng/mL CXCL8 (1 mM CaCl_2_ final nominal concentration). In Both pre-mixes CXCL8 significantly promoted stronger NETosis compared to their respective basal media. Statistical analysis: two-way ANOVA followed by Šídák's multiple comparisons test.

**Figure S7. Failure of various inhibitors to suppress CaCl₂- and CaCl₂ + CXCL8-induced NETosis.** Neutrophils were pre-incubated for 30 minutes with different inhibitors prior to stimulation with 1 mM CaCl₂ alone or combined with 100 ng/mL CXCL8. The inhibitors tested were: DPIC (NADH/NADPH oxidase inhibitor, panel **A**), CHIR99021 (GSK-3α/β inhibitor, panel **B**), iCRT3 (Wnt/β-catenin transcription inhibitor, panel **C**), rapamycin (mTOR inhibitor, panel **D**), lonafarnib (farnesyl transferase inhibitor, panel **E**), and PD98059 (MEK inhibitor, panel **F**). None of these compounds significantly inhibited NETosis under these conditions. Data represent three independent experiments. Statistical analysis was performed using two-way ANOVA followed by uncorrected Fisher’s LSD multiple comparisons test.
